# Proximal ganglionic intestine in Hirschsprung Disease is fibrotic and stiff

**DOI:** 10.1172/jci.insight.199820

**Published:** 2026-02-10

**Authors:** Prisca C. Obidike, Britney A. Hsu, Chioma Moneme, Oluyinka O. Olutoye, Walker D. Short, Mary Hui Li, Swathi Balaji, Yuwen Zhang, Sundeep G. Keswani, Lily S. Cheng

**Affiliations:** 1 Division of Pediatric Surgery, Department of Surgery, University of Virginia, Charlottesville, Virginia, USA.; 2 Department of Surgery, Baylor College of Medicine, Houston, Texas, USA.; 3 Division of Pediatric Surgery, Department of Surgery, Texas Children’s Hospital, Houston, Texas, USA.

**Keywords:** Gastroenterology, Neuroscience, Collagens, Fibrosis, Neurodevelopment

## Abstract

Hirschsprung disease (HSCR) is a congenital intestinal disorder characterized by the absence of ganglia in the distal intestine. Despite surgical resection of the aganglionic intestine and pull-through surgery, patients with HSCR still experience bowel dysfunction, indicating that latent abnormalities may also exist in the proximal ganglionic intestine. To elucidate possible causes of postoperative bowel dysfunction in HSCR, we investigated differences in the proximal ganglionic intestine using an animal model of HSCR (*Ednrb*-null mice) and validated our findings in tissue from patients with HSCR. We found that the proximal ganglionic colon of HSCR mice exhibited greater stiffness and fibrosis than their WT littermates. Similarly, submucosal fibrosis was significantly greater in the proximal ganglionic intestine of patients with HSCR than in intestinal tissue from age- and site-matched controls. Furthermore, we observed dysregulated expression of extracellular matrix–related (ECM-related) genes in the proximal ganglionic intestine of HSCR mice compared with controls. We conclude that increased fibrosis, stiffness, and alterations in ECM composition may contribute to persistent dysfunction of the ganglionic intestine in HSCR. These findings add to the growing body of literature that describe abnormalities in the proximal ganglionic intestine of HSCR and suggest that HSCR is not limited to the aganglionic intestine alone.

## Abstract 2

The proximal ganglionic intestine in Hirschsprung disease (HSCR) is fibrotic and stiff. These alterations in the extracellular matrix may contribute to the persistent bowel dysfunction that HSCR patients experience even after pull-through surgery.

## Introduction

The enteric nervous system (ENS) is a complex network of neurons and glia within the gastrointestinal (GI) tract that regulates gut motility and homeostasis independently of the central nervous system (1–4). The ENS is derived from enteric neural crest cells (eNCC), which migrate caudally from the vagal neural tube to colonize the developing gut (5, 6). Disruption of eNCC migration can lead to disorders such as Hirschsprung disease (HSCR) (7, 8). HSCR, a congenital GI disease that affects 1 in 5,000 live births, is characterized by a complete absence of ganglia in the distal intestine (2, 9). The current standard of care is surgical resection of the aganglionic intestine and pull-through of the ganglionic intestine (10, 11). However, even after successful surgery, more than half of patients experience persistent bowel dysfunction, including chronic constipation, fecal incontinence, and Hirschsprung-associated enterocolitis (HAEC), a leading cause of morbidity and mortality in this population (7, 11, 12). The persistence of bowel dysfunction despite surgical resection of the aganglionic intestine suggests that latent abnormalities may also be present in the proximal ganglionic intestine.

The GI microenvironment, particularly the extracellular matrix (ECM), plays a critical role in ENS development by guiding eNCC migration, proliferation, and differentiation along the GI tract (8, 13–15). The ECM, a 3-dimensional acellular structure composed of collagens, glycoproteins, and proteoglycans, provides structural support and biochemical cues essential for coordinated ENS development (8, 13). Alterations in ECM components such as laminin and collagen have been observed in ganglionic segments of HSCR intestine and may contribute to its pathogenesis (16, 17). Importantly, ECM composition also governs tissue mechanical properties, such as stiffness, which also influence eNCC behavior during development (18). Although ECM remodeling continues after birth (13), the ECM composition and biomechanical properties of postnatal HSCR intestine remain poorly characterized. To identify potential contributors to HSCR-related bowel dysfunction, we investigated ECM composition and mechanical properties in the proximal ganglionic intestine of HSCR. We hypothesized that both are altered in HSCR compared with normal intestine.

To test this, we used *Ednrb*^–/–^ mice, a widely accepted animal model of HSCR that exhibits distal aganglionosis of the colon similar to the most common phenotype of human HSCR (19–21). We assessed the mechanical properties and ECM composition of the proximal ganglionic intestine in these mice and validated these findings using resected bowel from patients with HSCR. To determine whether observed differences were due to disease-specific pathology or a consequence of bowel distention, we also analyzed distended intestinal tissue patients with intestinal atresia. Our findings contribute to the growing body of evidence suggesting that the proximal ganglionic intestine in HSCR is not entirely normal and may play a role in postoperative bowel dysfunction.

## Results

### The proximal ganglionic colon in HSCR mice is stiff and fibrotic.

**Figure 1:**
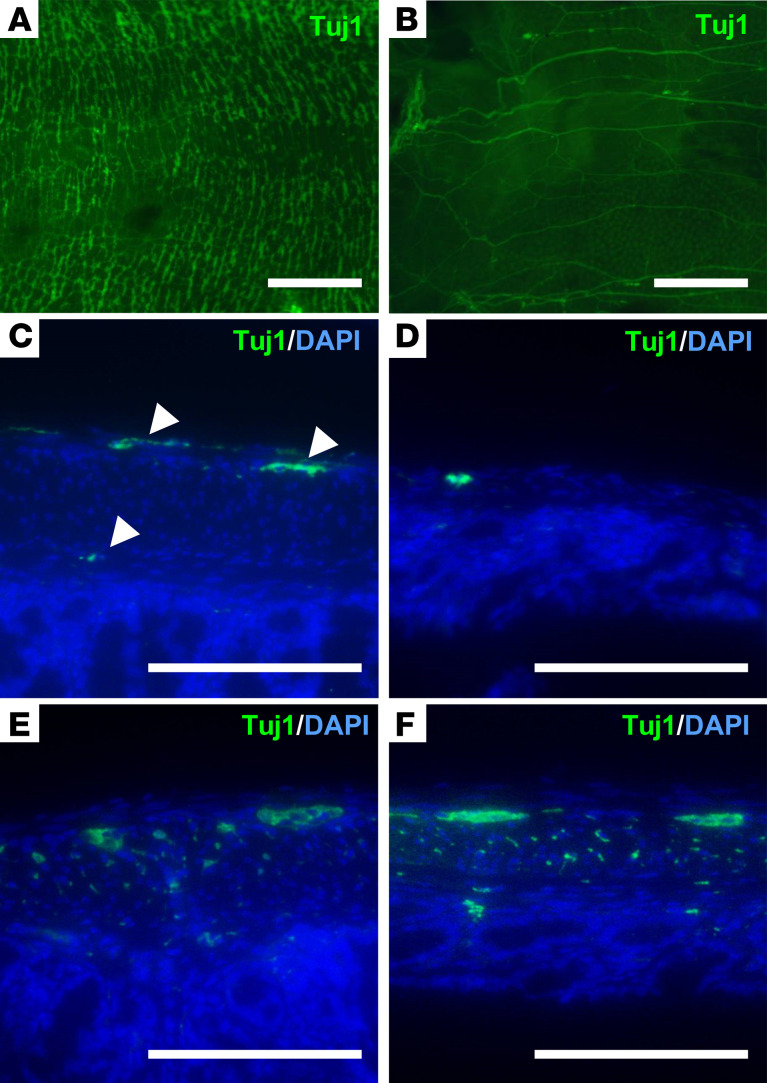
Reduced ganglion and neuronal density in the distal colon of HSCR mice. Aganglionosis of in the distal *Ednrb*^–/–^ colon was confirmed by IHC for the panneuronal marker, Tuj1. (**A** and **B**) Whole mount IHC of the muscularis propria demonstrated abundant innervation of the ganglionic segment (**A**) and sparse innervation of the aganglionic segment (**B**) in the *Ednrb*^–/–^ mouse. (**C** and **D**) Similarly, tissue sections of the proximal colon in the *Ednrb*^–/–^ mouse confirmed the presents of myenteric and submucosal ganglia (**C**; white arrowheads), whereas sparse and small ganglia are seen in the distal colon (**D**). (**E** and **F**) In contrast, equivalent segments of the proximal and distal colon in the WT (*Ednrb*^+/+^) littermate demonstrate abundant ganglia in both proximal and distal segments (**E** and **F**). Scale bar: 500 μm for **A** and **B**, and is 100 μm for **C**–**F**.

**Figure 2:**
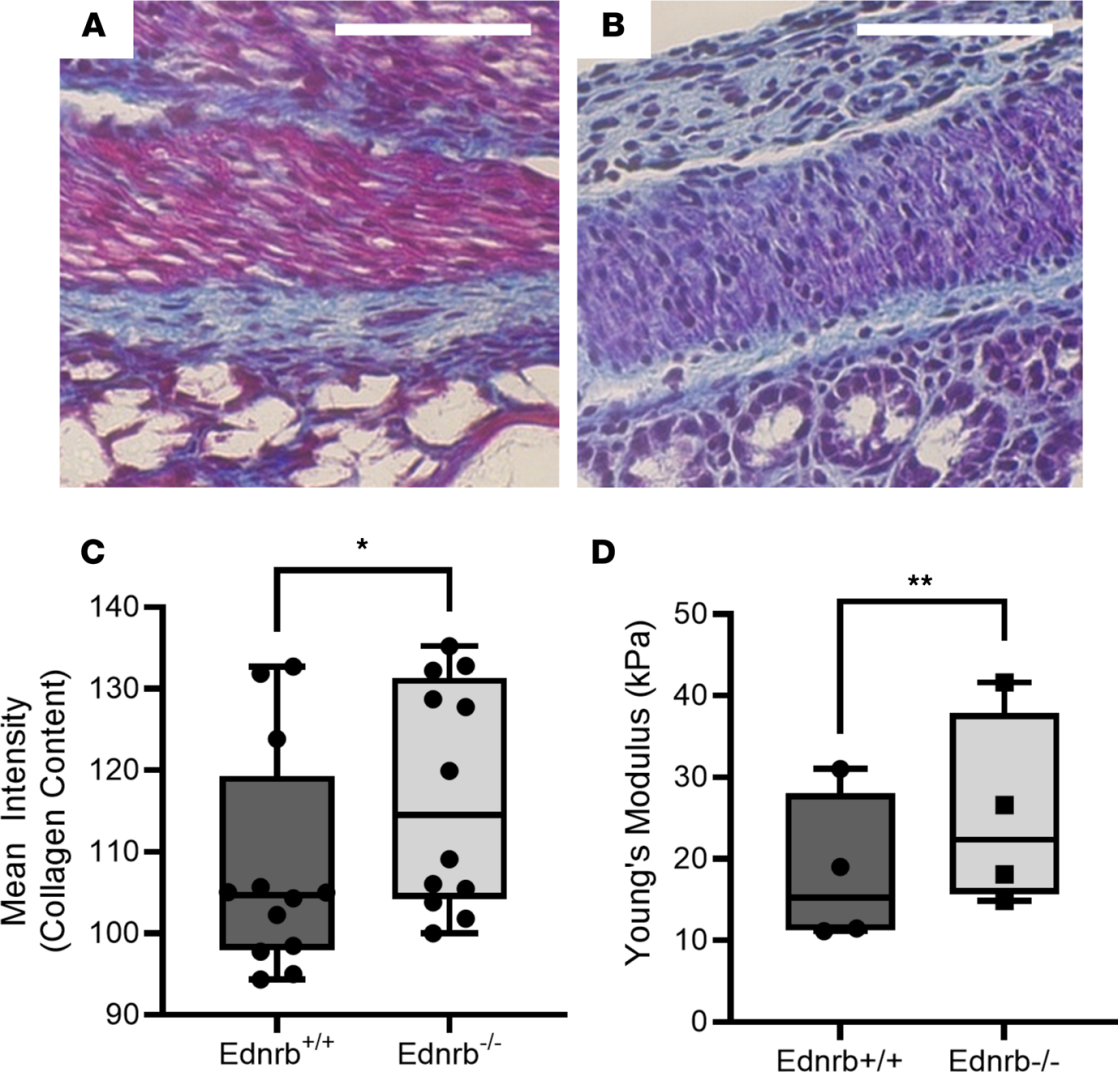
HSCR mice have stiff and fibrotic proximal ganglionic colon. (**A** and **B**) Representative images of the proximal ganglionic colon in WT (*Ednrb*^+/+^, **A**) and HSCR (*Ednrb*^–/–^, **B**) mice are shown with Masson’s trichrome staining to quantify fibrosis. Atomic force microscopy was used to quantify stiffness. (**C** and **D**) When normalized to age-matched WT littermates (*n* = 12), HSCR (*n* = 12) mice had significantly greater collagen content in the muscularis propria (**C**; *P = 0.006). HSCR (*n* = 4) mice also had significantly greater stiffness (**D**; **P = 0.0003) when compared with WT littermates (*n* = 4). Each data point represents the average of at least 5 fields of view of 1 biological replicate. Student’s 2-tailed *t* test used for statistical analysis. Scale bar: 100 μm for **A** and **B**.

Masson’s trichrome staining was performed on proximal ganglionic mouse colon to examine collagen content in the muscularis propria layer. Normoganglionosis was confirmed by IHC for the pan-neuronal marker, Tuj1 (Figure 1, A–F). Histological analysis of representative images showed greater collagen deposition, represented in blue by Masson’s trichrome staining, in the proximal ganglionic colon of HSCR mice (*n* = 12) compared with equivalent proximal colon segments of their WT littermates (*n* = 12) (Figure 2, A and B). Quantification of collagen deposition in both groups showed that HSCR mice had greater collagen content in the proximal colon than their WT littermates (116.92 ± 13.76 versus 108.04 ± 13.65, *P* = 0.006; Figure 2C). Similarly, the collagen content in the distal colon of the WT mice was significantly greater than the distal colon of HSCR mice (111.90 ± 10.83 versus 86.156 ± 11.32; *P* = 0.025).

Atomic force microscopy (AFM) was used to examine the stiffness of the colonic tissue from HSCR and their WT littermates. The elastic modulus, which represents the stiffness, is the force required to deform these tissues. The AFM cantilever probe was brought in contact with the muscularis propria layer as identified by microscopy*,* and the elastic modulus was obtained. We observed that the proximal colon of HSCR was significantly stiffer than that of WT mice (25.29 ± 11.96 versus 18.17 ± 9.31 kPa, *P* = 0.015; Figure 2D).

### Dysregulation of ECM-related genes in HSCR proximal ganglionic colon.

**Figure 3:**
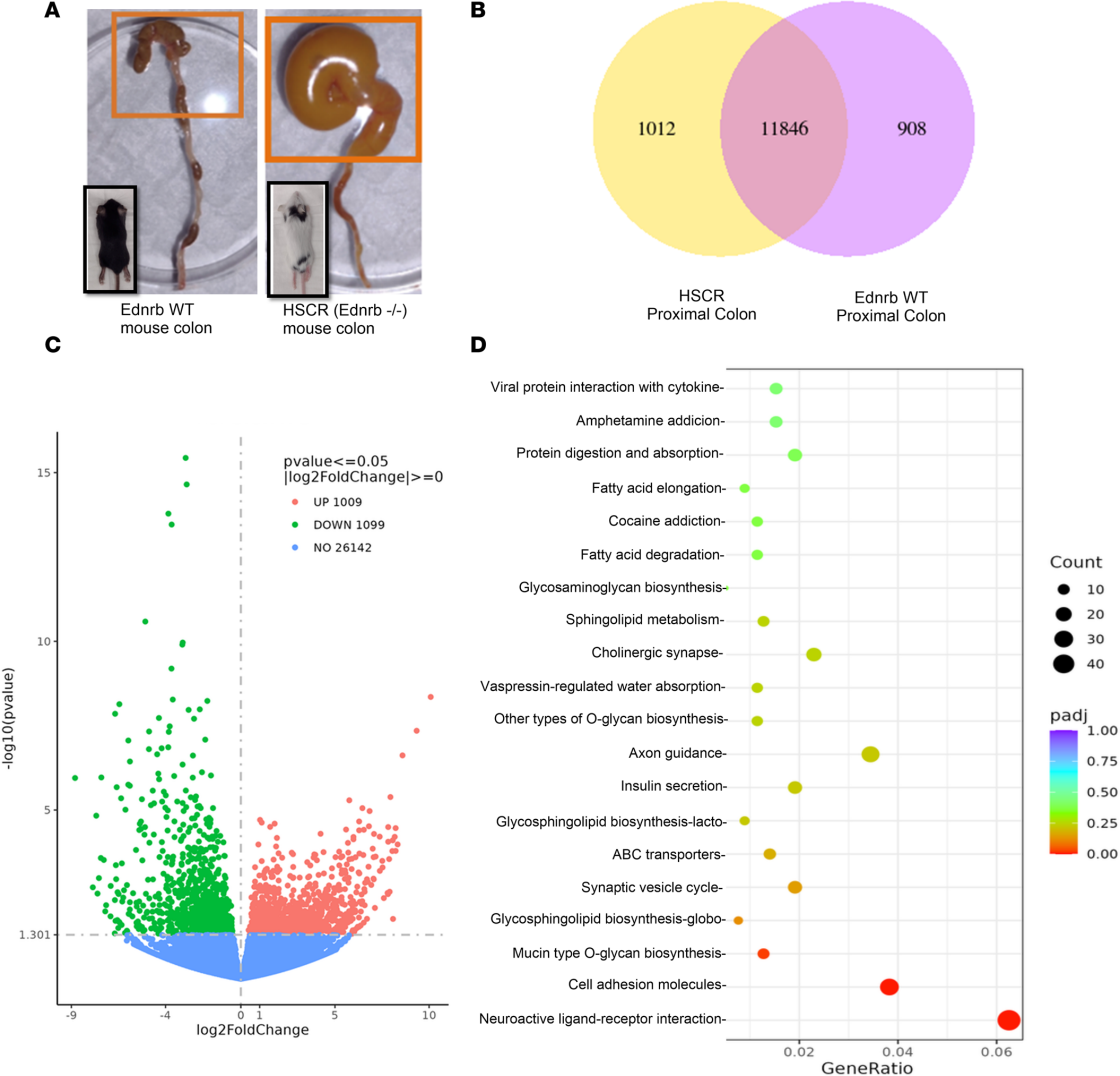
Differential gene expression in proximal ganglionic colon. (**A**) The longitudinal muscle and myenteric plexus from the proximal colons of HSCR (*Ednrb*^–/–^) and WT (*Ednrb*^+/+^) littermates were analyzed for differences in RNA expression. (**B**) Venn diagram comparing differentially expressed genes (DEGs) in the proximal colon of HSCR and WT mice identified 1,012 uniquely dysregulated genes in HSCR and 908 in WT with 11,846 genes in common between both groups. (**C**) Volcano plot of RNA-seq shows that 2,108 genes were differentially expressed with 1,009 upregulated genes and 1,099 downregulated genes. (**D**) Lollipop plot of top enriched KEGG pathways among DEGs.

Bulk RNA-seq was performed to examine the relative expression of ECM-related genes in the proximal colon of HSCR compared with WT mice (Figure 3A). RNA was extracted from homogenized LMMP samples obtained from the freshly dissected proximal colon and sequenced to construct a polyA enrichment library. Differential expression analysis demonstrated 1,012 genes uniquely dysregulated in the proximal colon of HSCR mice, 908 unique to WT littermates, and 11,846 genes shared between both groups (Figure 3B). A volcano plot comparing gene expression between HSCR and WT proximal colon identified

In total, 1,009 genes were upregulated and 1,099 genes were downregulated (log_2_FC > ± 1, *P*_adj_ < 0.05) (Figure 3C). Kyoto Encyclopedia of Genes and Genomes (KEGG) enrichment analysis revealed statistically significant differences in pathways related to neuroactive ligand-receptor interaction and cell adhesion molecules between HSCR and WT mice (Figure 3D).

**Figure 4:**
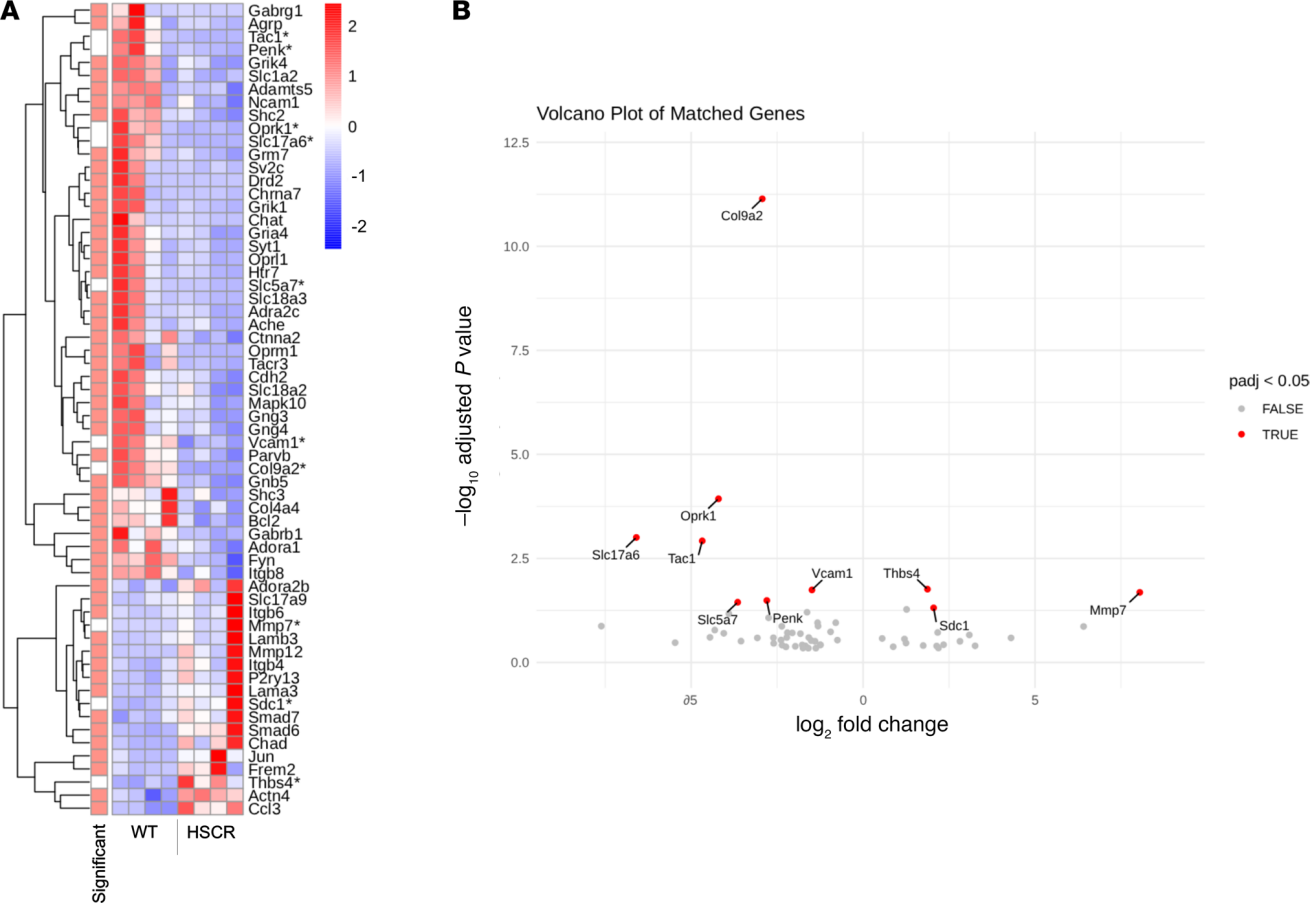
Extracellular matrix–related differential gene expression in proximal ganglionic colon. (**A**) Heatmap comparison of differentially expressed genes involved in focal adhesion, fibrosis, ECM-receptor interaction, and neuroactive ligand–receptor pathways in the proximal colon of HSCR and WT mice (*P_adj_ <0.05). (**B**) Volcano plot showing Log2FC of 10 statistically significant genes (*P*_adj_ < 0.05) involved in fibrosis and extracellular matrix.

We also queried differentially expressed genes specifically for genes involved in focal adhesion (22), ECM-receptor interaction (23), neuroactive ligand-receptor interactions (24), ECM (25), and fibrosis (26) (Supplemental Tables 1 and 2; supplemental material available online with this article; https://doi org/10.1172/jci.insight.199820DS1). Of the 62 individual genes in these pathways, 10 were significantly up- or downregulated (*P*_adj_ < 0.05) (Figure 4, A and B). Specifically, the proximal ganglionated colon of HSCR mice demonstrated increased expression of genes encoding Thrombospondin 4 (*Thbs4*), matrix metallopeptidase 7 (*Mmp7*), and Syndecan-1 (*Sdc1*). Downregulated genes included Vascular cell adhesion molecule 1 (*Vcam1*), Collagen type IX, α 2 subunit (*Col9a2*), Opioid receptor κ 1 (*Oprk1*), Solute carrier family (sodium-dependent inorganic phosphate cotransporter) member 6 (*Slc17a6*), Tachykinin 1 (*Tac1*), Preproenkephalin (*Penk*), and Solute carrier family 5 (choline transporter) member 7 (*Slc5a*) (Figure 4B).

### Ganglionic intestinal tissue of patients with HSCR is fibrotic.

**Figure 5:**
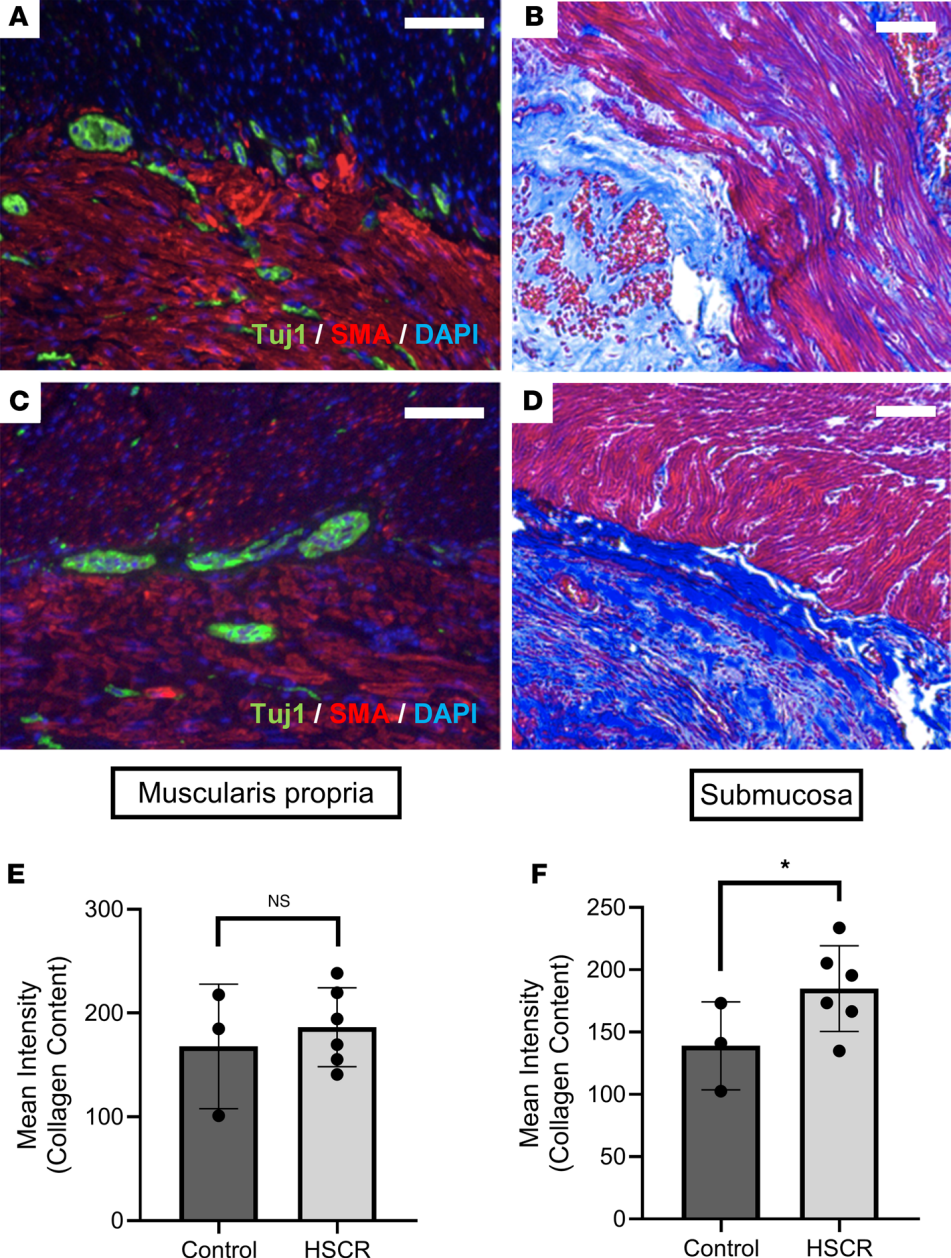
Patients with HSCR have increased fibrosis in ganglionic intestine. (**A** and **C**) Representative immunofluorescence images of the proximal ganglionic intestine in healthy control patients (**A**) and in patients with Hirschsprung disease (HSCR; **C**) confirms the presence of normal ganglion cells within the muscularis propria (Tuj1 is a pan-neuronal marker, SMA is smooth muscle actin, DAPI denotes cell nuclei). (**B** and **D**) Masson’s trichrome staining was used to compare collagen content between control patients (**B**) and patients with HSCR (**D**). (**E** and **F**) Patients with HSCR (*n* = 6) had increased fibrosis in the muscularis propria (**E**; *P* = 0.08) and significantly increased fibrosis in the submucosa (**F**; *P = 0.0002) when normalized to age- and site-matched controls (*n* = 3). Each data point represents the average of at least 5 fields of view of 1 biological replicate. Student’s 2-tailed *t* test used for statistical analysis. Scale bar: 100 μm for **A**–**D**.

To validate our findings in humans, we obtained intestinal tissue specimens from patients with HSCR (*n* = 6) and age-matched controls (*n* = 3) with no history of congenital colorectal disease who underwent bowel resection, ostomy formation, or ostomy closure (Table 1). Intestinal tissues were examined with Masson’s trichrome staining to quantify collagen content in the muscularis propria and the submucosa (Figure 5, A–D). Notably, the collagen content in the submucosa was significantly greater in the ganglionic intestinal tissue of patients with HSCR compared with equivalent segments from control patients (184.91 ± 34.30 versus 133 ± 27.02, *P* = 0.001; Figure 5, E and F). In contrast, there was no significant difference in the collagen content of the muscularis propria between HSCR and control patients (186.40 ± 37.92 versus 148.45 ± 53.14, *P* = 0.082).

### Comparison of fibrosis in HSCR, intestinal atresia, and ARM.

**Figure 6:**
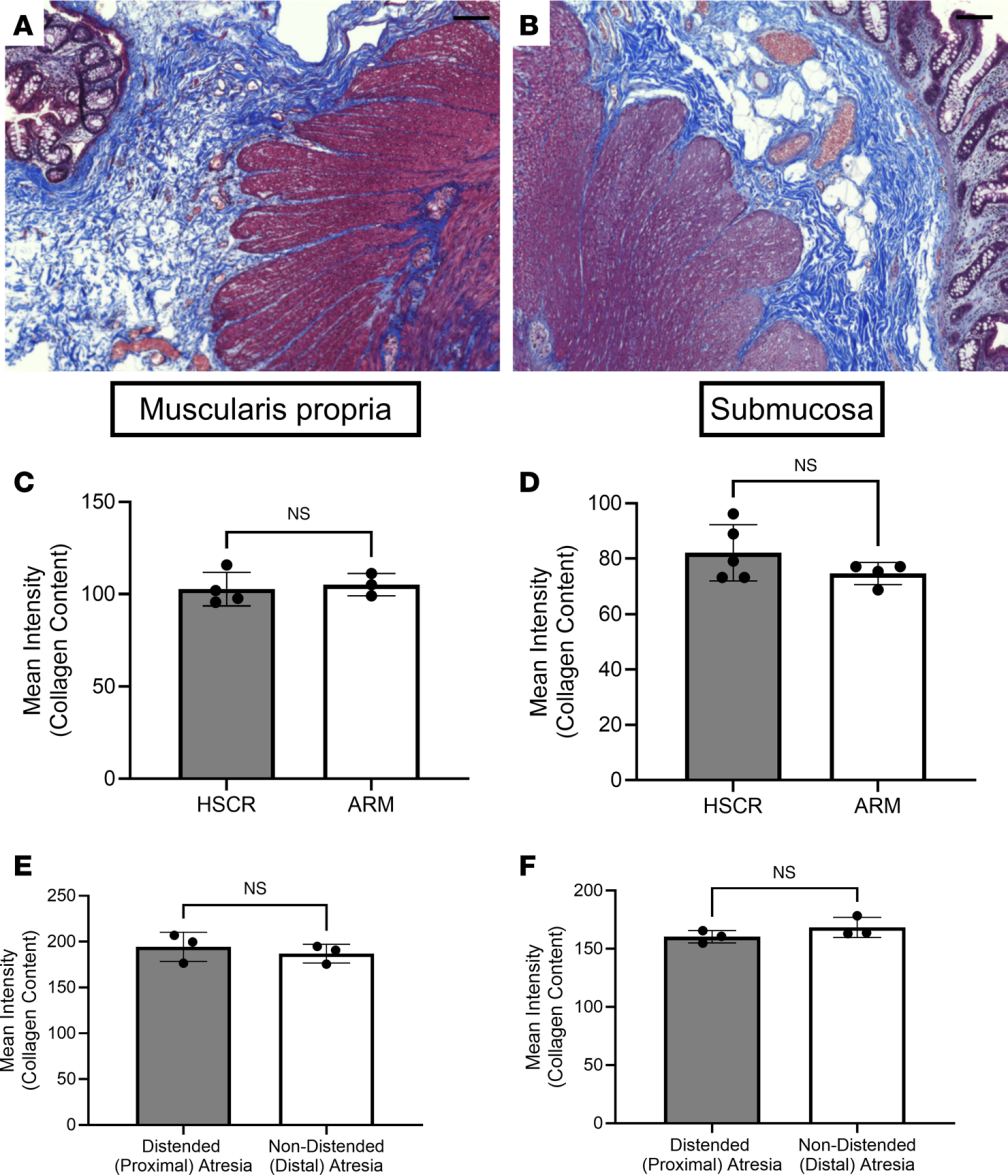
No difference in fibrosis in age-matched HSCR, ARM, and patients with intestinal atresia. (**A** and **B**) Representative Masson’s trichrome staining of proximal ganglionated intestinal tissues from age-matched patients with HSCR (**A**) and ARM (**B**). (**C** and **D**) No notable difference in fibrosis was observed between patients with HSCR (*n* = 4) and ARM (*n* = 3) in either the muscular propria or the submucosa. (**E** and **F**) Patients with jejunoileal atresia had no notable difference in fibrosis between distended proximal and nondistended distal intestinal segments (*n* = 3 each). Each data point represents the average of at least 5 fields of view of 1 biological replicate. Student’s 2-tailed *t* test used for statistical analysis. Scale bar: 100 μm for **A** and **B**.

To determine whether fibrosis in the proximal ganglionic colon in HSCR is due to disease-related pathology or secondary to chronic intestinal distention, we compared collagen content between distended proximal and nondistended distal intestine in patients with intestinal atresia (Table 1). There were no significant differences in the collagen content in either submucosa (168.42 ± 8.61 versus 160.43 ± 5.35, *P* = 0.103) or muscularis propria (186.99 ± 10.21 versus 194.43 ± 15.94, *P* = 0.154) of the distended and nondistended intestine in patients with intestinal atresia (Figure 6, E and F).

To further assess whether intestinal fibrosis was specific to HSCR, we quantified fibrosis in age- and site-matched tissues from patients with HSCR and ARM. Patients with ARM were chosen as they were the closest age and tissue match for younger patients with HSCR. No significant differences in the collagen content in either the submucosa (80.42 ± 12.39 versus 76.64 ± 6.82, *P* = 0.373) or the muscularis propria were observed (102.78 ± 9.08 versus 105.11 ± 6.02, *P* = 6286) in the HSCR compared with ARM tissues (Figure 6, A–D).

## Discussion

When Harald Hirschsprung first described HSCR in 1886, he said he believed that dilation of the proximal colon was the main cause of disease (27). Only a decade later did Karl Tittel recognize that HSCR was actually due to aganglionosis of the distal colon, leading to the surgical treatments that are the standard of care today (28). However, despite resection of the aganglionic intestine and pull-through of the ganglionated intestine, many patients with HSCR continue to experience bowel dysfunction. The cause of this dysfunction is not fully known, but suggests that underlying abnormalities exist in the proximal ganglionated intestine. Our results show that the proximal ganglionated colon is stiff and fibrotic with dysregulated ECM composition.

Previous studies have identified abnormalities within the ganglionated intestine in HSCR. Specifically, altered neuronal density, neurotransmitter expression, and dysmotility have been noted in the ganglionic region of the *Ednrb*^–/–^ mouse (1, 20). Similar findings have been validated in patients with HSCR and have been found to correlate with clinical outcomes (29, 30). Abnormalities in the microenvironment of the ganglionated colon have also been described. Deviations in ECM composition of the gut during development are noted to contribute the pathogenesis of HSCR (8, 16, 31, 32). Notably, increased expression of collagen VI has been found in the ganglionic colon of patients with HSCR, especially patients with HSCR with Down syndrome, and collagen VI is thought to interfere with normal eNCC migration to contribute to the pathogenesis of HSCR. An increased expression of collagen I and III and decreased expression of collagen IV in the proximal colon relative to the distal colon has also been described (14). In our study, we describe for the first time to our knowledge that overall collagen content, manifested as fibrosis, is significantly greater in ganglionated HSCR intestine compared with normal intestine. These findings add incremental knowledge to a growing body of evidence suggesting that the ganglionic HSCR intestine is not as normal as once believed.

The cause of fibrosis and ECM dysregulation in HSCR is not known, but we hypothesized that it may be secondary to chronic distention of the ganglionic region. Mechanical tension has been mechanistically linked to fibrosis in other organ systems (33–36). The ECM is considered a viscoelastic material that exhibits both viscous characteristics, evidenced by its ability to deform gradually without full recovery, and elastic properties, demonstrated by immediate deformation with the capability to return to its original shape when exposed to mechanical stress (35, 37, 38). Thus, the ECM surrounding intestinal cells remodels constantly to absorb the mechanical forces that occur during digestion and elimination. In the proximal ganglionated bowel, which is subject to significant distention in HSCR, the ECM may be altered to support tissues under greater mechanical stress. Interestingly, however, we noted no significant fibrosis in another model of intestinal distention: congenital intestinal atresia. This may be due to disease-specific differences in atresia compared with HSCR. In intestinal atresia, a vascular insult in utero disrupts intestinal continuity, leading to intestinal obstruction and proximal bowel distention, but ENS structure and function are normal (39). Altered wound healing and reduced fibrosis in the fetal environment in intestinal atresia may also contribute (40, 41). The absence of increased fibrosis in the distended segment proximal to the atretic area may also reflect that surgical repair typically occurs shortly after birth, as seen in the younger age of our patient cohort. Consequently, the brief duration of obstruction may not allow sufficient time for chronic tissue remodeling to develop. Another contributing factor may be the limited sample size and use of small intestine rather than colonic tissue for analysis. Our lab is currently developing a postnatal model of chronic intestinal distention to better determine if fibrosis is secondary to distention or specific to HSCR. Alternatively, fibrosis in the ganglionic HSCR intestine may be due to inflammation, such as HAEC (42), or prior surgery. While we did not explore the potential contribution of inflammation to intestinal fibrosis in this study, we note that no patients exhibited clinical signs of HAEC at time of bowel resection and no tissue specimens were taken from a previous surgical site. Still, prior surgery and enterocolitis could influence tissue stiffness and fibrosis. To determine if HAEC is associated with fibrosis, future studies might examine fibrosis and ECM composition in patients with and without a history of HAEC.

Fibrosis is often associated with increased stiffness (43, 44). Using AFM, we measured that the stiffness of the proximal ganglionated colon of HSCR mice was approximately 1.4 times greater than equivalent intestinal segment from WT littermates. AFM is considered the gold standard for measuring mechanical properties of soft biological samples due to its ability to detect nanomechanical forces and precise spatial resolution without tissue deformation (45, 46). Although there are numerous contributors to tissue stiffness, increased collagen content and collagen cross-linking are strongly associated with increased tissue stiffness (18, 47, 48). Our findings, and previous work by Chevalier et al. (18), support the understanding that collagen, the most abundant ECM component, is a key contributor to tissue stiffness in the GI tract. Stiffness, as well as collagen content, plays a crucial role in eNCC migration during development (18). The effect of postnatal tissue stiffness and fibrosis on the ENS remains to be seen and is the subject of ongoing studies in our lab.

Limitations of this study include a small sample size of patients with HSCR and controls. Thus, our study may be underpowered to detect a difference in fibrosis in the muscularis propria in human HSCR. Though each tissue specimen was matched for age and site, specimens encompassed a wide age range and varying intestinal locations. This was primarily due to the limited availability of appropriate controls. To eliminate potential confounding factors, we limited controls only to patients with no diagnosed GI or systemic disease, specifically, healthy patients who had intestinal resection for unexpected traumatic injuries. This resulted in an older cohort of patients with HSCR in whom fibrosis may be more pronounced due to chronicity of symptoms. We separately compared a cohort of younger patients with HSCR with age- and site-matched intestinal tissue from patients with ARM and found no difference in fibrosis; however, this may be due to confounding intestinal abnormalities in patients with ARM, who also experience chronic distention and dysfunction. Additionally, in younger patients (including patients with intestinal atresia), the absence of increased fibrosis in the distended segments may reflect an acute presentation with insufficient time for tissue remodeling to occur. Another important limitation is that nerve fiber hypertrophy, a key histological feature of aganglionosis, may vary with the extent of aganglionosis in both animal models and patients. This variation may contribute to differences in tissue architecture. In our animal studies, the small number of significant ECM-related genes identified could also be due to sample size and within-sample heterogeneity. Additionally, we used trichrome staining, a widely accepted technique for quantifying collagen content in tissues, but we did not explore the differences in specific collagen types as has been done in previous studies. Lastly, though we did not measure stiffness of the ganglia directly, previous studies have found that the stiffness of the muscularis propria closely represents the stiffness of the ganglia (46).

In conclusion, our study demonstrates that the proximal ganglionated intestine in HSCR is stiff, fibrotic, and exhibits dysregulated expression of ECM-related genes. These abnormalities may contribute to the postoperative bowel dysfunction observed in patients with HSCR.

## Methods

### Sex as a biological variable.

Our study included male and female animals and individuals. Similar findings are reported for both sexes.

### Animal subjects.

Male and female heterozygous *Ednrb^tm1Ywa^* mice on a hybrid C57BL6/J-129Sv background were purchased from the Jackson Laboratory and bred to obtain homozygous (*Ednrb*^–/–)^ mice and WT (WT; *Ednrb*^+/+^) mice. *Ednrb*^–/–^ mice, commonly used as a model for human HSCR, have a disrupted endothelin B receptor gene that results in distal colonic aganglionosis and a piebald coat color, while their *Ednrb*^+/+^ or ^+/–^ littermates appear phenotypically normal (21). Mice were genotyped by PCR to distinguish between *Ednrb*^+/+^ or ^+/–^ mice. Two- to 4-week-old mice were euthanized, and their entire colons were dissected and removed. The proximal ganglionic colon was delineated by gross colonic distention in *Ednrb*^–/–^ mice and confirmed by IHC for the panneuronal marker, Tuj1. Equivalent segments of proximal colon were collected from WT littermates.

### Patients.

Ganglionic intestinal tissues were collected from 2 cohorts of patients with HSCR to enable age-matched comparisons. (a) Equivalent segments of intestinal tissue were collected from normal age-matched control patients with no prior history of systemic or intestinal disease who were undergoing stoma formation or closure after traumatic injuries (Control: *n* = 3; range 2–13 years versus HSCR: *n* = 6; range 1–14 years old). (b) Additional samples were collected from patients with anorectal malformations (ARM: *n* = 4, range 3 weeks to 10 months old versus HSCR: *n* = 5; range 9 days to 2 years old).

Normoganglionosis was confirmed by histologic examination in all cohort tissues. Proximal and distal intestinal segments were also obtained from patients with intestinal atresia (*n* = 3; range 3 days to 2 months old) to compare fibrosis in distended and nondistended intestines without HSCR. The age and sex of all patients are listed in Table 1. This study was approved by the Baylor College of Medicine IRB.

### Histology and IHC.

Colonic tissues from mice and patients were isolated and fixed in 10% formalin and embedded in paraffin in a standard histological fashion. Tissues in paraffin-embedded blocks were sectioned at a thickness of 5 μm using an RM 2235 Microtome (Leica Biosystems) and placed onto glass slides. Tissue sections were then deparaffinized and rehydrated to PBS following standard protocol, and IHC staining was performed on a Dako Auto-stainer Link 38 (DakoLink version 4.1, edition 3.1.0.987; Agilent). Tissue sections were permeated with 1% BSA and 0.1% Triton X-100 in PBS for 1 hour, before being incubated with the primary antibody, mouse anti-Tuj1 (1:500; GTX130245**,** GeneTex) at 4°C for 24 to 48 hours. Then, tissues were washed in PBS and incubated with a secondary antibody, rabbit anti–mouse Alexa Fluor 488 (1:1,000; A27023, ThermoFisher) at 4°C for 1 hour. Slides were mounted using a DAPI-containing mounting media (VectaShield Anti-fade mounting media; Vector Laboratories).

For H&E staining, tissue sections were stained using the SelecTech H&E system (Leica Biosystems) according to the manufacturer’s instructions. For trichrome staining, tissue sections were stained using Masson’s trichrome special stain kit (Leica Biosystems) according to the manufacturer’s instructions. Images were taken using a Leica DM500 or DMi8 microscope (Leica Biosystems).

### Quantification of collagen content.

Collagen content was quantified using trichrome-stained tissue sections. Collagen content was calculated using the color threshold tool in ImageJ (NIH) according to previously published methods (11). For animal studies, the average collagen content was calculated from representative images of the colonic muscularis propria in the proximal and distal colon for *Ednrb*^–/–^ mice and WT littermates (*n* = 12 per group). The average collagen content for each *Ednrb*^–/–^ mouse was normalized to its WT littermate control for statistical comparisons. For patients, the average collagen content per patient sample was calculated separately from representative images of the muscularis propria and the submucosa for patients. For patients with HSCR, each sample was normalized to the closest age- and site-matched control and patient with ARM. For patients with jejunal-ileal atresia, the proximal intestinal collagen content was normalized to the distal intestinal collagen content for the same patient.

### Quantification of tissue stiffness.

The BioScope II AFM (Bruker Corporation) was used to quantify the mechanical stiffness of the proximal colon tissues using a colloidal probe technique. Tissues were embedded in OCT media, flash frozen at –80°C, cryosectioned at a thickness of 10 μm, and mounted onto a poly-L-lysine-coated glass slide. A Nikon TE2000 microscope confirmed the probe’s location within the muscularis propria layer. A 2.5 μm spherical colloidal probe on a soft cantilever (k = 0.24 N/m) was used to indent tissue samples by 4 μm at 0.5 Hz. The elastic modulus of each tissue specimen was obtained by fitting the force-indentation curve using a Hertz model. The elastic modulus for each *Ednrb*^–/–^ mouse was normalized to its WT littermate control for statistical comparisons.

### RNA extraction and bulk RNA-seq.

The longitudinal muscle and myenteric plexus (LMMP) were isolated from proximal and distal colonic segments by microdissection of *Ednrb*^–/–^ mice and their WT littermates (*n* = 4 each group). Tissues were homogenized, and RNA was isolated using the RNeasy Kit (Qiagen), and bulk RNA-seq was performed. The resultant RNA-Seq data were mapped using HISAT2 to the mouse genome build UCSC mm39. Gene expression quantification was achieved using FEATURECOUNTS (49) against the GENCODE gene model. Differential gene expression was evaluated using the R package EdgeR (50) and was further normalized using the R package RUVr4 (LRT RUVr). Significance was defined as a fold change exceeding 1.5× and FDR < 0.05. Volcano plots were generated using the Enhanced Volcano package in the R statistical system. Enriched pathways were assessed via the over-representation (ORA) (51) method using the hypergeometric distribution and the MSigDB (16) v7.5.1 genesets. Additional gene ontology (GO) term analyses were performed using ShinyGO (52).

### Statistics.

Data are represented as mean ± SD. Statistical analysis was performed using GraphPad Prism version 9.0 for Windows (GraphPad Software) and R (version 4.4.1). Statistical significance of collagen content and tissue stiffness was compared using a 2-tailed unpaired Student’s *t* test. We considered a *P* < 0.05 to be statistically significant.

### Study approval.

All mice were housed in a controlled environment with a 12-hour light/dark cycle and provided access to food and water ad libitum. All animal-based experiments were approved by the IACUCs of Baylor College of Medicine and the University of Virginia.

### Data availability.

Data generated or analyzed are included in the Supporting Data Values file. Sequencing data can be accessed using accession no. GSE305622.

## Author contributions

PCO, BAH, CM, OOO, WDS, MHL, YZ, and LSC performed experiments, analyzed data, and interpreted results of experiments. SB, SGK, and LSC conceived and designed research. PCO, BAH, CM, and LSC prepared figures and drafted the manuscript. All authors edited, revised, and approved the final version of the manuscript. BAH and PCO share first authorship based on their equal contribution to this project. First authorship was based on the amount of work contributed to the experiments, data collection, analysis, and manuscript preparation.

## Conflict of interest

The authors have declared that no conflict of interest exists.

## Funding support

This work is the result of NIH funding, in whole or in part, and is subject to the NIH Public Access Policy. Through acceptance of this federal funding, the NIH has been given a right to make the work publicly available in PubMed Central.

- NIH/NIDDK K08DK133673 (to LSC)
- Texas Medical Center Digestive Diseases Center P30DK056338 (to LSC)
- American College of Surgeons Franklin H. Martin Faculty Research Award (to LSC)
- American Pediatric Surgical Association Jay Grosfeld Scholar Award (to LSC)
- NIH/NIGMS R01GM111808 (to SGK)
- NIH/NHLBI T32HL007849 (to PCO and CM)

## Supplementary Material

Supplemental data

Supplemental table 1

Supplemental table 2

Supporting data values

## Figures and Tables

**Table 1:**
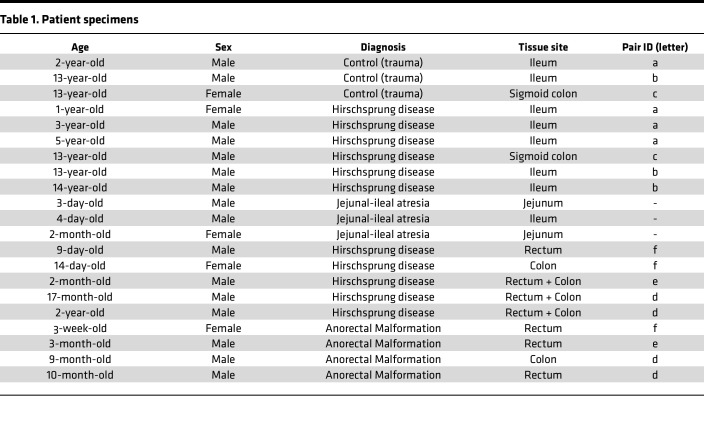
Patient specimens

## Version 1 02/10/2026

In-Press Preview

## Version 2 07/08/2026

Electronic publication
