# Supplementary material for: Proximal ganglionic intestine in Hirschsprung Disease is fibrotic and stiff
Source: JCI Insight. 2026 Feb 10;11(13):e199820. doi: 10.1172/jci.insight.199820 (PMC13460888; doi:10.1172/jci.insight.199820)
Supplement: Supplementary file 1 [file jciinsight-11-199820-s215.pdf]

SUPPLEMENTAL MATERIAL

Supplemental Table 1:

List of all differentially expressed genes in the proximal colon of wildtype and HSCR littermates. DOI: 10.25833/bdr0-kf36

Supplemental Table 2:

Genes involved in fibrosis, ECM-receptor interaction, focal adhesion, and neuroactive-receptor ligand pathways in Mus musculus (house mouse) (22–24) that were queried for heat map shown in Figure 4. DOI: 10.25833/bdr0-kf36
